# Turning a blind eye and a deaf ear to traditional and complementary medicine practice does not make it go away: a qualitative study exploring perceptions and attitudes of stakeholders towards the integration of traditional and complementary medicine into medical school curriculum in Uganda

**DOI:** 10.1186/s12909-018-1419-4

**Published:** 2018-12-18

**Authors:** Amos Deogratius Mwaka, Gervase Tusabe, Christopher Orach Garimoi, Sunita Vohra

**Affiliations:** 1 Department of Medicine, School of Medicine, College of Health Sciences, Makerere University, P.O Box 7072, Kampala, Uganda; 2 Cancer Awareness and Early Detection Coalition (CAEDCO), Kampala, Uganda; 3 Department of Philosophy, School of Liberal and Performing Arts, College of Humanities and Social Sciences, Makerere University, Kampala, Uganda; 4 Department of Community Health and Behavioural Sciences, School of Public Health, College of Health Sciences, Makerere University, Kampala, Uganda; 5 Departments of Paediatrics, Medicine, and Psychiatry, University of Alberta, Edmonton, Canada; 6 Faculty of Medicine & Dentistry, Integrative Health Institute, University of Alberta, Edmonton, Canada

**Keywords:** Biomedicine, Traditional and complementary medicine, Traditional medicine, Therapeutic alliance, Traditional health practitioners

## Abstract

**Background:**

A substantial proportion of healthcare professionals have inadequate understanding of traditional and complementary medicine and often consider their use inappropriate.

**Methods:**

We conducted a qualitative study to understand the perceptions and attitudes of medical students, medical school faculty and traditional and complementary medicine practitioners. In-depth interviews and focus group discussions were used to collect data. Thematic approach was used in data analysis to identify emerging themes and sub themes. Data analysis was supported with use of Atlas.ti v6.1.1.

**Results:**

The majority of participants commended the inclusion of traditional and complementary medicine principles into medical school curricula. The main reasons advanced were that: patients are already using these medicines and doctors need to understand them; doctors would be more accommodating to use and not rebuke patients, thereby minimizing delays in care due to pursuit of alternative therapies; promote patient safety; foster therapeutic alliance and adherence to therapy; uphold patients' right to self-determination; lead to discovery of new drugs from traditional medicines; and set ground for regulation of practices and quality control. However, participants anticipated operational and ethical challenges that include inadequate number of faculty to teach the subject, congested curricula, increased costs in research and development to produce evidence-base data, obstruction by pharmaceutical companies, inaccessibility to and depletion of medicinal plants, and potential conflicts due to diversity in culture and values. A substantial minority of participants thought traditional medicine need not be taught in medical schools because there is lack of scientific evidence on efficacy, safety, and side effects profiles. These shortfalls could make the determination of benefits (beneficence) and harm (maleficence) difficult, as well as compromise the ability of physicians to adequately disclose benefits and harms to patients and family, thereby undermining the process of informed consent and patient autonomy.

**Conclusions:**

Training medical students in principles of traditional and complementary medicine is considered reasonable, feasible, and acceptable; and could lead to improvement in health outcomes. There are anticipated challenges to implementing a hybrid medical school curricula, but these are surmountable and need not delay introducing traditional and complementary medicine principles into medical school curricula in Uganda.

**Electronic supplementary material:**

The online version of this article (10.1186/s12909-018-1419-4) contains supplementary material, which is available to authorized users.

## Background

Worldwide, the use of traditional and complementary medicine (T&CM) has increased exponentially especially in the treatment of chronic illnesses including cancers [1]. In the US, more than half (54% of 604) of adult cancer patients initiated use of complementary therapies after cancer diagnosis in the hospitals, mainly for improvement of general health status and quality of life [2]. In Japan, cancer patients (44.6% of 3100) were significantly more likely to use herbal medicines compared to patients with other benign conditions (25.5% of 361) [3]. In sub Saharan Africa, about 60–70% of people use T&CM for various illnesses including mental illness and sexually transmitted diseases [4, 5]. In East Africa, a recent report revealed that up to 70% of people first consult traditional healers before they visit biomedical facilities [6]. Traditional and complementary medicine practices are common in Uganda across a range of illnesses and people use them for various reasons including beliefs in intrinsic efficacy, long history of use, and perceived barriers to biomedical care [7–10]. It is likely that use of T&CM will continue side by side with western medicines [11]. In addition, the World Health Organization (WHO) traditional medicine strategy 2014–2023 stipulates the importance of integrating traditional medicine practices into mainstream biomedical practices. The WHO developed the strategy with the aims to keep populations healthy through supporting Member States to develop and implement proactive policies and action plans, and to provide guidance on regulatory and quality standards in order to ensure safety, efficacy and quality use of T&CM. The strategies on traditional medicines also aim to promote rational use of T&CM as well as increase access to the poor populations through improved availability and affordable quality T&CM regimens [12].

The ethical principle of autonomy demands that human agents who are considered to be adults and legally competent decide what is right for themselves including their healthcare choices. However, patients and healthcare professionals need evidence to guide decisions in order to prevent harms. The duty to prevent harm demands that healthcare professionals know about the potential benefits and harms of both conventional medicine and T&CM, and provide timely information to their patients and the public. General knowledge regarding the theories and principles of T&CM practices could help physicians in guiding patients about their health choices [13]. Healthcare professionals need to balance the ethical obligations to maximize good and avoid harm to their patients with the evidence of effectiveness and safety of both T&CM and conventional therapies. It is now known that awareness of T&CM and careful ethical considerations are necessary to provide holistic care for patients [14]. There are, however, limited data on the nature of the ethical dilemma regarding the autonomy of patients who wish to use T&CM and the benevolent tendency (paternalism) of healthcare professionals towards safeguarding the health and wellbeing of their patients from perceived dangers of T&CM. This potentially conflicting obligations and rights claims may stifle integration of T&CM and biomedicine both at the medical school curricula development and practice levels.

Including T&CM in medical school curricula increases awareness and knowledge of the future clinicians (medical students) on T&CM, and potentially fosters cooperation and collaboration between T&CM and biomedicine [15]. Training medical students on T&CM theories and practices enables the students understand better the paradigm from which traditional health practitioners treat their patients [16]. Accordingly, many academic institutions in the high-income countries (HICs) including Harvard, Duke, Yale, and Stanford universities have introduced integrative medicine training in their medical school curricula. In the US, an analysis of websites for 130 medical schools for courses in complementary medicine in the medical school curriculum showed that half of the schools (50.8%) offered at least one course or clerkship in complementary medicine. The most frequently reported instructional methods were lectures, readings, and observation [17].

In Uganda and most sub Saharan African countries, there is limited data on medical students’ specific needs for and attitudes towards T&CM education. In this study, we explored the attitudes and perceptions of medical students and their lecturers at Makerere University College of Health Sciences (MakCHS), and traditional health practitioners regarding the inclusion of T&CM theories and principles into medical school curricula in order to inform integration of T&CM and biomedical practices in Uganda.

## Methods

### Study design and setting

This was a qualitative study in which we employed in-depth interviews (IDI) and focus group discussions (FGD) approaches to collect data from medical students, lecturers and traditional medicine practitioners. No repeat or follow up interviews/discussions were carried out. This study was conducted at the School of Medicine, Makerere University College of Health Science (MakCHS) and Kampala district, the capital city of Uganda. Makerere University School of Medicine which started in 1923 is the oldest medical school in Uganda and the East African region. There are several traditional health practitioners in Kampala. These practitioners have associations under which they operate. Majority of the practitioners have received training in various aspects of traditional practices and conventional medicine from THETA Uganda, headquartered in Kampala.

### Study population and period

Participants to this study included medical students and their lecturers, and practitioners and leaders of traditional health practitioners resident in Kampala. We included registered second, third, fourth and fifth year medical students, and lecturers in the three selected departments of internal medicine, paediatrics and psychiatry in the service of the university during the study period. We chose these departments based on our experiences of high prevalence of use of T&CM by patients who attend care in there. We also included representatives of the current leadership of the traditional health practitioners in Kampala during the study period. The study was conducted during April and May 2017, a time when the University was open and the medical students were preparing for the university examinations starting in mid-May.

### Sampling and recruitment of participants

Purposive sampling technique [18] was employed for participants of focus group discussions (FGDs), and in-depth interviews (lecturers and traditional health practitioners). The selection of the students’ FGDs was facilitated by the president of the medical students association. The lecturers were identified with support of their heads of departments. The executive director of THETA identified the overall leader of the traditional health practitioners, through whom other practitioners within Kampala district were subsequently identified. A list of 12 T&CM practitioners was made. Each practitioner on the list was asked to identify other practitioners. Practitioners on the list who were mentioned by an already interviewed practitioner were the only ones interviewed, a form of a snowballing approach, whereby an identified participant identifies another potential participant who is then approached by the research team [19–23].

The research assistants were trained for 2 days on the study objectives, sampling procedures, recruitment and consent procedures, and basic information regarding use of complementary and traditional medicines (T&CM) and the World Health Organization (WHO) recommendations regarding incorporating traditional medical practices into mainstream biomedicine.

The study was introduced to the students in their respective lecture theatres following mobilization. Explanations about the purpose and objectives of the study were provided to all eligible participants before enrolment. Thereafter, the participants were given opportunity to ask questions regarding the study. We then provided detail information on the study including objectives, inclusion criteria, consent procedures and rationale for selection of the specific category of participants to those who accepted to participate. There were no students who declined participation. Participants for FGDs registered their names, age, and year of study. They provided verbal consents to participate in the study. Additional verbal consent was sought to audio-record the discussions.

### Sample size estimation

Sample size for FGDs and IDIs was determined through the data saturation approach for qualitative research, whereby interviews and discussions were conducted until additional two to three interviews/discussions would yield no new information/themes [24]. FGDs were categorized by sex and year of study. We categorized participants by sex because traditional and complementary medicines are commonly used for management of gendered issues including poor erection/manhood, perceived inadequate vaginal fluid during sexual intercourse, and slow labour progress that would probably not be freely discussed when both sexes are together. There were four FGDs for male and three FGDs for female students from second and fifth years. Three lecturers were interviewed from each of the departments, ensuring there is at least a female participant from each department. Five traditional health practitioners were purposively included in the study (Table 1). We used FGDs for the students because the method can generate a large amount of data on an issue in a very short span of time and at affordable costs. In addition, the social interactions and group dynamics in FGDs exposes shared experiences, and allow for critical evaluations of views to avoid challenges from members of the group [25–27]. On the other hand, we used in-depth interviews with the faculty and traditional health practitioners because the method can produce rich, detailed accounts from the perspectives of individual respondents [28]. We intended to collect detailed accounts of participants’ thoughts, attitudes, beliefs, experiences and knowledge pertaining to use of and need for including complementary and traditional medicines in medical school curricula and the national health system. These could be well done using in-depth interviews [28–30].

**Table 1:** Participants characteristics

Participant Identification code	Gender	Category	Age/age category (years)	Data collection methods
P1, FGD_2nd Years, Female	Female	2nd years	20–25	FGD
P2, FGD_2nd Years, Male_1	Male	2^nd^ years	21–24	FGD
P3, FGD_2^nd^ Years, Male_2	Male	2^nd^ year	21–24	FGD
P4, FGD, 5th Years, Female_1	Female	5th years	23–26	FGD
P5, FGD_5th Years, Female_2	Female	5th years	24–26	FGD
P6, FGD_5th Years, Male_1	Male	5^th^ years	24–26	FGD
P7, FGD_5^th^ Years, Male_2	Male	5^th^ years	24–26	FGD
P8, IDI, Senior Lecturer, Male	Male	Faculty, Paediatrics Department	Not disclosed	IDI
P9, IDI, Herbalist_4, Female	Female	T&CM Practitioner	46	IDI
P10, IDI, Herbalist_5, Female	Female	T&CM Practitioner	Not disclosed	IDI
P11, IDI, Associate Professor, Female	Female	Faculty, Medicine Department	38	IDI
P12, IDI, Lecturer, Female	Female	Paediatrics Department	Not disclosed	IDI
P13, IDI, Senior Lecturer, Female	Female	Psychiatry Department	Not disclosed	IDI
P14, IDI, Herbalist_1, Male	Male	T&CM Practitioner	40	IDI
P15, IDI, Herbalist_2, Male	Male	T&CM Practitioner	Not disclosed	IDI
P16, IDI, Herbalist_3, Male	Male	T&CM Practitioner	Not disclosed	IDI
P17, IDI, Senior Lecturer, Male	Male	Faculty, Medicine department	40	IDI
P18, IDI, President, Male	Male	3rd year Student	23	IDI
P19, IDI, Senior Lecturer, Male	Male	Faculty, Paediatrics Department	Not disclosed	IDI
P20, IDI, Professor, Male	Male	Faculty, Psychiatry Department	Not disclosed	IDI
P21, IDI, Senior Lecturer, Male	Male	Faculty, Psychiatry Department	Not disclosed	IDI

*T&CM* traditional and complementary medicine, *IDI* in-depth interviews, *FGD* focus group discussion

### Data collection

The tools for the study were developed based on experiences of the investigators and discourse on traditional medicine and perceptions regarding inclusion of traditional and complementary medicines into medical school curricula. The study tools were piloted with the respective category of participants before use in data collection. The research assistants participated in piloting the study tools under supervision. The tools were refined based on the pilot data and a final tool was developed following consensus between the investigators. The three research assistants were university graduates in education and social sciences.

The president of the medical students association in conjunction with the class representatives and research assistants made appointments with the students. They formed groups by year of study and sex. Time was scheduled for each of the planned FGDs. Two research assistants conducted the FGDs. One research assistant was in charge of recording and taking field notes while the other guided the discussions and ensured participations by all group members. There were 6–10 members in each group. Appointments were also made with the university lecturers, president of the medical students’ association and traditional medicine practitioners for in-depth interviews. Interviews and focus group discussions were carried out in convenient locations including individual lecturer’s office. No non-participants were allowed in the interview/FGD venues. Data were collected using semi-structured tools/study guides (Additional files 1 and 2). Proceedings of the interviews/discussions were audio recorded. Field notes including nonverbal communications were taken to augment audio recorded information and ensure completeness of data.

### Data management and analysis

Audio recordings from the FGDs and IDIs were transcribed within 1 week of interview/discussions by one of the research assistants when memory of the interactions were fresh in the mind. Transcribed data were reviewed by first author and any incomplete segments were completed through listening to the respective audio recordings. Cleaned data sets were exported into ATLAS.ti version 6.1.1 for qualitative data analysis. Transcripts from FGDs and IDIs were analysed together using the thematic approach based on codes formulated from the themes in the study tools and those emerging from the data itself. Analysis codes were formulated by ADM and a medical anthropologist at department of psychiatry, Makerere University. Variations in code definitions were resolved through discussions between ADM, the medical anthropologist and the investigators. The codes were used to identify meaning segments from each of the transcripts. The meaning segments were aggregated and used to develop themes and subthemes. Representative quotes exemplifying each themes were captured in the results section.

## Results

### Study respondents

The study participants comprised nine university faculty (lecturers and professors), medical students (seven FGDs of preclinical and clinical years) and five practising traditional health practitioners. The age of the university faculty ranged between 36 and 49 years, while the medical students’ age range was 23–26 years. The traditional health practitioners were mainly herbalists and spiritualists aged 35–50 years. All the faculty and traditional health practitioners were married, while only two male students reported being married. The two married students were previously clinical officers.

The university lecturers had been faculty at the university school of medicine for periods ranging between five and seventeen years. We included three (one female) lecturers from each of departments of medicine, psychiatry and paediatrics (Table 1).

### Introduce traditional and complementary medicine into medical school curricula

The majority of respondents expressed the need for immediate introduction of traditional and complementary medicine (T&CM) theories and principles into the medical school curricula in Uganda. Most of the faculty respondents thought that the process of integrating T&CM into medical school curricula is long overdue. However, participants from a minority of student FGDs thought that policy guidelines and further research to adduce evidence on safety and side effect profiles are needed before introduction. The reasons advanced for teaching theories, principles and practices of T&CM to medical students included the fact that T&CM are already being used and will perhaps continue to be used, fostering of therapeutic alliance, promoting patients’ rights and autonomy, encouraging prompt health seeking behaviour, and providing an opportunity for new drug discoveries. The views of the respondents did not significantly vary by gender. However, the preclinical students seemed to have less reservations regarding inclusion of T&CM into the curricula compared to the fifth year students. Results from FGDs are reported by year of study and sex to show apparent variations by these categories.

#### Use of traditional and complementary medicine is a reality and should not be ignored

The majority of respondents described personal and observed experiences from childhood through to interview time that attest to extensive use of T&CM under various circumstances by majority of people in Uganda including the healthcare professionals and high ranking political leaders and policymakers. Not including T&CM on the curricula is therefore seems a futile attempt to dismiss a reality.

*“I also agree that we should bring the complementary medicine into our undergraduate study . . . I agree with that because many people are diverting from modern medicine and are already using the complementary medicines”, (P2, FGD_2* ^*nd*^ *Year, Male).*

Introduction of T&CM into the curricula was viewed as an opportunity to validate what is already being used based on beliefs and long tradition of use.

*“Yes, they should introduce it in the curriculum because most of the patients we receive if you ask them . . . Most of them have at least tried it (T&CM) before coming to the hospital. So it would be important if we could study them (T&CM) and help the patients understand that such and such a medicine (T&CM) has been approved to manage such and such a condition”, (P4, FGD_5* ^*th*^ *Year, Female).*

All the faculty respondents except one (male) concurred that T&CM needs to be urgently introduced into the medical school curricula so that healthcare practitioners learn both conventional medicine and T&CM.

*“I think that should have been done ages ago, yeah. We’ve delayed; these should have come long time ago”, (P13, IDI, Senior Lecturer, Female).*

The teachers of the medical students (Faculty), particularly from departments of psychiatry and paediatrics were concern with the glaring absence of T&CM from the medical school curricula.

*“I think it is a good idea because the population we deal with, the patients we deal with, T&CM is embedded in people’s lives. We offer the modern medicine but in our practice and experience, patients still run away or they combine, or they start from the traditional and complimentary then come to us when it is worse”, (P19, IDI, Senior Lecturer, Male).*

Awareness of T&CM and the need not to pretend regarding the widespread use of T&CM by the same patient populations that come to the biomedical facilities has been emphasized by the faculty members. This fact is considered a good reason to teach T&CM in the medical schools in order to empower the healthcare professionals to deal with matters of T&CM use.

*“People need to be aware that this treatment option (T&CM) exists. For centuries people lived on these things, so there is no need to dismiss it. They may not have lived longer, but they lived on these things . . . I think the teaching has been that it should be dismissed. But it is also true that the majority of people in this country access this kind of treatment especially in the field of mental health, where if people feel unwell mentally most people don’t go to a psychiatric hospital. They go to the traditional healers who give them something; they either give them some herbs or give them some counselling. So introducing it in the curriculum is one step to get the students to know”, (P21, Senior Lecturer, Male).*

#### Promote prompt health seeking behaviour and foster therapeutic alliance

Most respondents contented that patients would readily discuss use of T&CM with their clinicians if they expect no rebuke for use of T&CM. An atmosphere where patients freely discuss their use of T&CM without rebuke from the clinicians potentially promotes prompt health seeking behaviour at biomedical facilities and fosters therapeutic alliance, thereby encouraging patients to take treatments and advice of the clinicians.

*“Those using T&CM believe that it helps them and at times it entails understanding such beliefs. So it will be easier working with them if we study T&CM. You can’t tell someone stop using this and you can’t tell them why because you don’t know. . . I am sure the patients just want to know why they should or shouldn’t use it and its side effects, or a better way they should utilize it. Actually if you know the side effects and you tell them, for example that you will actually get like liver issues or other side effects, it will be easy for them to feel guided”, (P4, FGD_5* ^*th*^ *Years, Female_1).*

Turning a deaf ear and a blind eye to the concerns of the patients and simply dictating to them what to do could jeopardise the patient-doctor relationship and lead to nonadherence with prescriptions and clinic appointments.

*“So, by us turning a deaf ear and also turning away from traditional and complementary medicine does not make it cease to exist; it does not make people stop using them. It is actually like a kid that is in the middle of the road and it is going to be knocked by a car and then you look away. You get what I mean? So the only way we can help our patients is by understanding what they are taking and trying to help them accordingly, not by distancing ourselves from what they take and assume we are helping them. The Chinese have actually welcomed it, studied it and actually are using it; so why can’t we do it here?” (P6, FGD_5* ^*th*^ *Years, Male_1).*

#### Improve patient safety

Safety to patients could be improved once doctors are knowledgeable about T&CM.

*“I do agree with introducing traditional medicine in the medical curriculum of Uganda. I believe that when we finish our undergraduate school we shall be interacting with people who are using this kind of medicine, so we need to know what it really contains; does it contain active compounds? So we can avoid giving drugs which can interact with it and cause bad side effects”, (P2, FGD_2* ^*nd*^ *Years, Male_1).*

#### Promote autonomy and right to self-determination

Patients are autonomous agents who have the right to determine what should and should not be done unto themselves whether in health or illness kingdom. The majority of respondents were particularly concern about respect of patients’ choice to use T&CM alone or in combinations with conventional medicines. The respondents argued that doctors need to study these T&CM in order to understand them and therefore respect the choices of their patients while offering meaningful supportive advice sensitive of sociocultural beliefs and values.

*“These drugs are already in use and we should also know that it makes the backbone of modern medicine . . . as doctors we should agree that they are taught. We talk of the principle of respect of autonomy, so we should agree that there are other drugs other than ours and we should support its use”, (P3, FGD_2* ^*nd*^ *Years, Male_2).*

*“Whether you like it or not our patients do use these T&CM; it is their choice. It is like faith; if you feel this cannot work, the other can work and you cannot take that away from them. So the idea is actually to train doctors who understand these two aspects of medicine, the traditional and modern medicine. I think if it is introduced and the medical doctors get to understand what they do and how they can complement modern medicine, I think this can be a good idea for both the patients and the doctors because some of them actually do work”, (P6, FGD_5* ^*th*^ *Years, Male_1).*

#### Gateway to quality control and discovery of new medicines

The majority of respondents (medical students and faculty) mentioned that the inclusion of T&CM into medical school curricula is a correct step in the path towards understanding traditional health practices. Quality control and safety in traditional medicine practices could be easier to achieve in an integrated health system.

*“Just imagine if the Europeans were not bold enough to explore and carry out research on their medicine; would we have the so called biomedicine today? Of course not! So, let us not be closed minded, let’s introduce these things and study about them into details, you never know it will promote research and innovation in them . . . because studying them will help us to know the effects of these drugs to the body and these will enhance the way we treat people who are taking herbal medicine. At the moment it is just a green area, controlled by the traditionalist and not very well regulated”, (P2, FGD_2* ^*nd*^ *Years, Male_1).*

*“So I would say that it is important to bring them in the curriculum . . . it shouldn’t just be to discuss them but it should be linked to testing some of these herbs. Some of them have active ingredients that could be a basis for some of these modern drugs. So maybe some of the many herbs that we have if tested can be improved . . . if they are tested maybe we could have more discoveries that are going to deal with issues that low income countries are struggling with”, (P8, IDI, Senior Lecturer, Male).*

The traditional health practitioners were in addition concern with setting up regulatory framework and methods of collaborations before fully introducing complementary and traditional medicines into medical school curricula. They estimated that such phased approach could ensure equitable distribution of benefits and avoid conflicts between the biomedical and the traditional health systems.

*“I think it should go through processes whereby we first of all gain the capacity; first of all we harmonize the two systems whereby they appreciate each other, and respect should be put in place. So we should build on the background of the two systems in order to come up with a common position”, (P16, IDI, Herbalist_3, Male).*

### When to teach T&CM to medical students

There was general consensus that T&CM theories, principles and practices should be taught throughout medical school and postgraduate training. Respondents suggested that theories and principles could be taught to the preclinical students together with the other basic biomedical sciences including pharmacology, physiology and biochemistry. Respondents proposed that the clinical students (fourth and fifth years) could be made to visit the traditional health practitioners in their practices so as to understand the details of what the traditional medicine practitioners actually do.

*“Medical practitioners will need as much information on T&CM as possible and to grasp it, just like it takes over five years to study medicine it could as well take some time to comprehensively study T&CM. So studying it alongside the other course units from first year will be a good thing”, (P2, FGD_2* ^*nd*^ *Years, Male_1).*

*“In my opinion I think it will have to be a continuous process; you introduce it in first year, then continue with it in second year and third year. By the time you are in fourth year and fifth year you are able to integrate it into whatever you are doing”, (P5, FGD_5* ^*th*^ *Years, Female_2).*

The faculty had similar view that T&CM should permeate the whole curricula from first year to fifth year.

*“Right from the start, otherwise if you delay people will not understand. So it is better to begin introducing it in stages. There is when we send our students to communities -that would be a good place also to integrate that aspect in, so that when they go to communities and they are interfacing with people in communities they ask, they interview, they integrate”, (P19, IDI, Senior Lecturer, Male).*

There were alternative views from the two male fifth years FGDs regarding when to start teaching T&CM to the undergraduate students. They were concerned about blurring the knowledge boundaries and confusing the principles of conventional medicines with T&CM theories and principles. They recommended that T&CM be introduced at fourth and fifth years when the students will have had firm grasp of the principles of conventional medicine.

*“I would think that maybe fifth year. In the other years you are still trying to grasp the concept of medicine and you can’t compare the efficacies of herbal drugs without knowing the medical drugs we are using. I think we should first let the students grasp the concept of conventional medicine. Students may get confused; at the beginning some of them are still naïve . . . so bombarding them with this herbal medicine stuff yet they came to medical school to study modern medicine is going to be challenging to their mental faculties”, (P6, FGD_5* ^*th*^ *Years, Male_1).*

*“I think this course unit cannot be taught in the early years of medical school, because it is going to take people off course. It should be in the later years but in recess (Laughter) . . . But when you put it as a course unit in first year, in first year people are eager to study the Biochemistry . . . but when you bring it in later, these people have had some experiences, their perception will not be biased”, (P7, FGD_5* ^*th*^ *Years, Male_2).*

### Do not include traditional and complementary medicine into the curricula

One fifth year students’ FGD and one faculty member (both male) were against the inclusion of T&CM theories and principles into the medical school curricula in Uganda. The major concerns were lack of scientific evidence on efficacy, safety and side effects profiles.

*“I think my duty is to discourage them because we don’t know how those herbs work. Why do you want to combine it with modern medicine? The principles are different, modern medicine is based on science, now this traditional medicine where you go and pull leaves from there, how do you combine it. This traditional medicine, it can’t be combined but it can coexist. Get traditional healers, train them, let them do their thing and let us continue doing our thing . . . People can be taught, people who want to do traditional medicine, they can be taught”, (P17, IDI, Senior Lecturer, Male).*

A substantial minority of members of one male fifth year FGDs suggested that T&CM could be taken as electives and or taught as a separate course by doctors at their will. These members also expressed unawareness of any standards to use to evaluate and monitor both the teaching and measure performance of students in the traditional and complementary medicine course.

*“Introducing this (T&CM) may not be very wise, because a lot of this traditional medicine has not yet been proven in a rational way like the way research is done in a rational way. So in the event that we introduce this in the curriculum what will be the standard against which we will be measuring them? I think enlightening the students about it is a good thing because patients are using it. However, teaching the theories, and principles, I don’t see if it adds any value”, (P6, FGD_5* ^*th*^ *Years, Male_1).*

### Anticipated challenges and ethical concerns regarding T&CM in the curricula

The majority of respondents who supported the notion of including T&CM into the medical school curricula in Uganda mentioned some ethical and operational concerns that require keen attention during implementation of the hybrid biomedical – traditional medicine curricula. The challenges discussed included lack of qualified teachers, increased financial costs, cultural diversity and terminology difficulty, inaccessibility to and depletion of medicinal plants, unfavourable attitudes, inequitable distribution of benefits and patent concerns, lack of enabling policies at practice level, resistance by pharmaceutical companies, inaccessibility to information and teaching resources, time constraints in existing curricula and safety concerns.

#### Few faculty to teach traditional and complementary medicine

Both the preclinical and clinical year students were concern that there are very few faculty who have the interests, knowledge and skills in T&CM theories, principles and practices. A reasonable proportion of the students expressed discomfort with the traditional medicine practitioners coming to teach the theories of T&CM in the lecture theatres in the medical schools. The students expressed concerns that these practitioners, most of whom have not undergone formal education, do not understand the contents of their medicines and would be unable to articulate what they practice.

*“Finding the manpower to teach T&CM will also be a challenge if it is going to be implemented across all the medical schools in the country”, (P2, FGD_2* ^*nd*^ *Years, Male_1).*

*“I wouldn’t mind introducing this idea of T&CM; my worry is who would teach it. When you bring herbalists to teach it, he would spread his gospel, he would want you to join him, he will convince you and we would end up having perverted doctors”, (P7, FGD_5* ^*th*^ *Years, Male_2).*

The medical student leader expressed deep concern with teachers of T&CM in the medical schools.

*“May be something that I see that can also bring headache is to get people who can teach; it may take time to acquire all that information. I don’t think those people that we have now that are practicing it are able to pass their information to other people, because I saw it with my aunt . . . even if another person asked, she cannot explain it. She has it in the head that this heals this condition and that is all . . . So the problem can be the time it will take for such people to learn and be able to pass on their knowledge to the medical students”, (P18, IDI, Student Leader, Male).*

#### Congested curricula

The students felt that the current curriculum is already too congested to accommodate any more subject matters including traditional and complementary medicine.

“*I wonder how they are going to manage squeezing T&CM into an already congested curriculum. We shall have students who don’t have time to do anything else, from class or wards to the library, nothing else (Laughter)”, (P2, FGD_2*^*nd*^ *Years, Male_1).*

*“The major challenge I see about introduction of T&CM into our curricula is unless you are going to increase the number of years someone is going to be in medical school! I don’t see how we shall create time for it”, (P7, FGD_5* ^*th*^ *Years, Male_2).*

The faculty and traditional medicine practitioners did not have any concerns with time constraints. The faculty espoused that the T&CM course would be integrated within the framework of existing courses such as pharmacology and biochemistry.

#### Uncertainty about benefits and harms of traditional and complementary medicine

The lack of scientific evidence to demonstrate that T&CM are good and will not cause troublesome effects to the patients directly or through interactions with conventional medicines has been a great ethical concern to the majority of participants. Both the students and faculty expressed varying degrees of ethical concerns with including T&CM in the medical school curricula at the moment.

*“My concern in terms of ethics are really in terms of safety, because we do not know what is in those drugs (T&CM), we don’t know how much active ingredients, we do not know how much to give, we don’t know the side effects! So how safe are we in giving them to the patients? And if we are to include them in the curriculum, what shall we say about safety? We need to study, go back to the baseline, study the safety of some of them, the effects on the body then we can start to include them in the curriculum”, (P12, IDI, Associate Professor, Female).*

*“You are talking about autonomy; patients deciding for themselves, but what if patients decide to take poison! We don’t have research about them, we don’t have background knowledge about them, so the only way they can be introduced is after background check has been done about these drugs (T&CM); to find out what they can or can’t do, that is the only place for them in medical school. It would be very contentious to give what we are not very sure about, and therein lies the ethical issue, because you cannot prescribe something that you are not very sure about. It wouldn’t be very right to train me on something that is not evidence based”, (P6, FGD_5* ^*th*^ *Years, Male_1).*

*“I believe introducing this will be harmful to our patients because very little is really known about these drugs . . . these things are not standardized . . . and besides we are supposed to inform our patients about everything we know about the drugs so that they make an informed decision but then we can’t with herbs . . . little is known about them”, (P3, FGD_2* ^*nd*^ *Years, male_2).*

Informed consent requires complete disclosure of benefits and harms to the patients and family and then allow them to decide on the course of care they so desire. When the medical professionals have inadequate knowledge about T&CM, the principle of right to self-determination and informed consents could be undermined.

*“I believe introducing this will be harmful to our patients because very little is really known about these drugs . . . we are supposed to inform our patients about everything we know about the drugs so that they make an informed decision but then we can’t with herbs and I believe little is known about them”, (P3, FGD_2* ^*nd*^ *Years, Male_2).*

Most of the respondents concurred that the population definitely perceive of some benefits from using the T&CM, otherwise they would not use them. However, these benefits just like the harmful effects have not been documented to allow teaching the same to the medical students in a systematic way.

*“The issue is people do not know what pharmacological content is in the herbs and when it is best suited, so that is where the problem is . . . for most of this traditional drugs they have not approved and documented the benefits. There is no evidence that there is a benefit of the drug (T&CM). And another thing, I have seen a lot of patients coming to the hospital and they have certain side effects out of these drugs. So even the harmful effects of these drugs haven’t yet been documented”, (P5, FGD_5* ^*th*^ *Years, Female_2).*

#### Delay in health seeking

The faculty member who objected to including T&CM into medical school curricula reasoned that acknowledging T&CM by virtue of inclusion into medical school curricula and perhaps integration into the national health system could potentially lead to patients taking long time with the traditional medicine practitioners, thereby reporting late for diagnosis and treatment at biomedical facilities.

*“Once it is fully accepted, people will think now I can fully hang around with the traditional and complementary medicine and in that way the patients may be delayed because they think that this is also an acceptable form . . . it will cause delays, it will affect patients’ adherence to treatment (biomedical), adhering to visits, and reviews”, (P19, IDI, Senior Lecturer, Male).*

#### Increased costs of medical training

Most members from both preclinical and clinical year FGDs were concern with the additional cost of hiring qualified faculty, research to develop evidence base, and the additional costs that students may have to part with to enable acquisitions of resources for T&CM teaching and especially practical sessions.

*“I am thinking it will not be cost effective, it may take years. We may have to have a basis to include them. So that calls for more research into it that will take time and resources. We have to show evidence that actually they have some use . . . So that will call for more expenditures”, (P1, FGD_2* ^*nd*^ *Years, Female).*

*“Like my colleague has suggested . . . Most of the data that is available is about herbs that we don’t have here. So we need to study our own medicine before we publish them to generate information that is unique to us, so it will take much longer for it to start”, (P4, FGD_5* ^*th*^ *Years, Female_1).*

#### Obstruction by pharmaceutical companies and private health practitioners

A faculty and the student leader were concern that pharmaceutical companies selling conventional medicines could be potential adversaries who would oppose development of evidence for T&CM and frustrate legislation and policies on T&CM.

*“In the bigger scheme of things, fights with the industries, for example industries making X (a conventional medicine); they may not be very supportive of these things. They can even malice you without you knowing, because you are cutting into their sales and that becomes problematic. Also some medical workers who are doing private practice may also have some trouble . . . Healthcare professionals could flatly reject the introduction of such ideas”, (P21, IDI, Senior Lecturer, Male).*

*“I see a number of challenges because as per now we have a number of pharmaceutical companies around and they are manufacturing modern medicine. They use herbals to manufacture modern medicines, so it may not be of a good market if traditional medicine is introduced into medical school . . . they will fight against it”, (P18, IDI, Student Leader, Male).*

#### Cultural diversity and harmonization challenge

There are diverse cultural groups in Uganda. Each of these cultural and ethnic groups have their unique traditional medicines for the varying illnesses. The names and constituents of the medicines also vary between the cultural groups and yet the students from all these diverse groups are together in the same medical schools. Respondents were concerned with cross cultural issues regarding the names and the particular traditional medicines to be prescribed in each circumstance.

*“I also believe most of these drugs are society related; the drugs used in Buganda (central Uganda) are not the same as those used in the North, so we will end up having a lot of . . . I don’t know, because we are posted to work in different regions, you are not going to tell me this is Ankole medicine in Buganda; it will make it so hard”, (P3, FGD_2* ^*nd*^ *Years, Male_2).*

*“The herbs that people use in Buganda maybe different from the herbs that people use in Lira; maybe different from the herbs which people use in Acholi, so I think introducing it in the curriculum is a very good suggestion, but then how to integrate the whole will be much harder. You find that maybe people in Buganda use Mutza (altered for purposes of protection) for fever yet someone else uses something else for fever . . . Everyone has their own concoction which makes it difficult to harmonize. They are too many drugs, it’s like in Buganda alone, there are like a hundred herbs, then those other regions, actually a hundred plus”, (P5, FGD_5* ^*th*^ *Years, Female_2).*

The faculty shared similar concerns regarding cultural diversity and harmonization of T&CM principles and practices.

*“Of course it would be a challenge in how these are defined. You know when you talk about these there is a lot of diversity, one culture will define it differently, one tribe will define it differently”, (P19, IDI, Senior Lecturer, Male).*

#### Conflict with personal values and religious beliefs

Conflicting values based on culture and religious beliefs and practices of the practitioners has potential to affect learning outcomes. Students with strong religious leaning may find it difficult to grasp the principles of traditional and complementary medicine which apparently have been demonised over time by the religious groupings in the country.

*“Yeah, if you bring a witchdoctor to class, you don’t expect a born again student to be in that class”, (P5, FGD_5th Years, Female_2).*

*“Actually the biggest problem would be the student’s attitudes; it would be so hard. I would give an example of myself, I don’t think I would sit in a class and somebody is talking about herbs. So, I don’t know how that is going to be addressed but I see that it is going to be a major challenge”, (P6, FGD_5* ^*th*^ *Years, Male_1).*

The faculty were equally convinced of a potential conflict based on religious grounds.

*“We are also of different faiths and spiritual backgrounds . . . when it comes to the spiritual it will be very contradictory. Some students may not be comfortable to undertake T&CM to certain depth because of their faith. Even the doctors, nurses and other health practitioners may have value conflict when it comes to prescribing traditional medicines”, (P19, IDI, Senior Lecturer, Male).*

#### Inaccessibility and depletion of medicinal plants

The traditional medicine practitioners were particularly concern with how the medical students will gain access to the medicinal plants. These respondents were equally concern with the manner in which the medicinal plants are being depleted without replenishment. These would make the medical students miss the practical exposure. In addition, once T&CM is adopted and taught in the medical schools, the doctors would start prescribing them. The supply of the medicinal plants therefore need to be increased, without which, patients would find difficulty accessing prescribed traditional medicines.

*“There are challenges of ensuring availability of natural resources to meet the demand, because at the moment people are not growing yet the demand is high. We are using plants, plants are endangered; there are so many people who disturb the plants, who misuse them. Then people are not planting; they just exploit and don’t replace. Another challenge is these plants are not known to communities; that they are useful in certain ways. You can find a very good plant because it is so large, now people of timber and those of charcoal or wood fuel they want to cut it down. They are not so easy to replace because some of them take so many years to develop”, (P16, IDI, Herbalist_3, Male).*

#### Intellectual property rights and patent

Most of the traditional medicine practitioners have their own concoctions of medicines. They derive financial gains and livelihoods from their practices. They are therefore concern that once their medicines have been tested and found useful, they should be rewarded or given patents for their unique products. Lack of acknowledgement could make the practitioners refuse to disclose the identity of their medicinal plants.

*“And then we should look at all aspects; people don’t know the issue of intellectual property rights. And then how are they going to preserve and conserve the knowledge”, (P16, IDI, Herbalist_3, Male).*

Two faculty members also shared similar concerns regarding intellectual property rights and patents.

*“We also have to convince the knowledge owners because most of them have the fear that ‘my knowledge will be taken yet it is my source of income’”, (P21, IDI, Senior Lecturer, Male).*

#### Limited credible resources on traditional and complementary medicine

The majority of respondents noted that the available published scientific evidence are mainly from China, Malaysia and other countries from South East Asia and the high income countries. Teachers of T&CM and medical students are expected to face insurmountable challenges in seeking for credible sources of information on T&CM.

*“The challenge I foresee, it all comes down to information. If you look at fields like nutrition you know the books have been written . . . there is very little database on Ugandan herbs. So first we may need to build up a data base which may take lots of years to set up”, (P7, FGD_5* ^*th*^ *Years, Male_2).*

A lot of the information about traditional medicines are got from informal sources including the traditional medicine practitioners and grandparents. There are challenges accessing information from these sources; for example, cultural norms require that elders are not questioned, while herbalists are overly protective of their information because the practice is a source of livelihood and they would not want anyone to just get the information about the particulars of their medicinal products.

*“There is no clear information; even the people who use these drugs (T&CM) and tell others to use it, they just know about the beneficial effect but they can’t explain how it works. There is no specific website, there is no book about that. Even when you find it, it is questionable . . . And in our culture, when you are of a certain age, you are not allowed to ask certain questions, so you can’t go to my grandmother and say I want to know how this thing works”, (P5, FGD_5* ^*th*^ *Years, Female_1).*

*“The herbalists are so protective of their medicine, you only receive it in bottles or jerry cans without clear formulas for fear of duplication by someone else. They also don’t explain into details about their work”, (P2, FGD_2* ^*nd*^ *Years, Male_1).*

*“We know these drugs (T&CM) are a source of money to these people who administer them, so they prefer to monopolize them so they can earn more. For me I grew up in a village so I know what happens. So normally its only one person who knows it in the village. They just come back with the drug from the bush and all the others don’t know his source”, (P7, FGD_5* ^*th*^ *Years, Male_2).*

In spite of the various challenges regarding teaching traditional and complementary medicine in the medical schools, the majority of respondents maintained that turning a deaf ear to and or looking away from the T&CM debate does not make matters any better since people will continue to use these remedies in their crude forms which may present greater danger to human health.

## Discussion

We have found that the primary stakeholders in medical school training and education (medical students and lecturers) as well as the traditional and complementary medicine practitioners consider introducing traditional and complementary medicine (T&CM) training into the medical school curricula in Uganda as necessary and important for the advancement of health outcomes in the population that often uses T&CM. Several reasons were advanced for including T&CM into the medical school curricula; foster understanding of the healthcare professionals of an already existing practice about which doctors know very little, promote prompt health seeking and therapeutic alliance, improve patients’ safety through research into T&CM and increased awareness about benefits and side effects, promote patients’ autonomy and right to self-determination, open up a gateway to new drug discoveries, and institute a quality control measure for traditional medicine practices. The respondents acknowledged some operational and ethical challenges that lie in the way of teaching T&CM to medical students. Rather than hone the challenges and turn eyes away from the fact of T&CM use, the respondents to this study contended that overcoming the challenges and maximizing benefits from teaching T&CM to medical students is a preferred alternative with long term benefits for population health. Training the future healthcare professionals (medical students) would enhance the integration of the two apparently independent health systems (traditional health system and biomedicine) into one functional and well-regulated national health system as is recommended by the WHO [12]. The integration of T&CM practice and biomedical system has been slow and only achieved in a few countries [31, 32]. The slow integration could be explained by a plethora of reasons, including low knowledge and perhaps negative attitudes of biomedical trained healthcare professionals and policymakers. At the international level, studies show that majority of physicians have limited knowledge concerning T&CM although most of them wish to know more about T&CM including evidence of their effectiveness and side effect profiles [33, 34]. Physicians who lack information about T&CM cannot engage fruitfully with patients regarding T&CM use and may castigate patients for using T&CM based on perceived side effects.

We found that use of T&CM is a reality that can no longer be denied without harms to the patients and health system. Respondents reported that most patients present at biomedical facilities after having used T&CM for their symptoms. Attempts by healthcare professionals to discourage use of T&CM has in the experience of our respondents simply undermined development of therapeutic alliance and created parallel healthcare systems that talk less to each other, thus undermining benefits from synergy and mutual cooperation. In most circumstances, help-seeking decisions about sources of care when illness strikes are guided by the people’s culture, belief systems and the interpretations of the meaning of illnesses [35]. In this regard, traditional medicine practitioners are therefore accepted and have remained central in health services because they provide care within the patients’ cultural context and belief system [36]. Alternate engagement with biomedical and traditional health systems or parallel use of T&CM and biomedicine perhaps contribute to delay in seeking biomedical care if patients choose to start their help-seeking journeys with the traditional medicine practitioners and only go to biomedical facilities thereafter. A study among breast cancer patients in Indonesia showed that use of traditional medicine before biomedical health seeking and after cancer diagnoses led to delay and advanced stage cancers at diagnoses and missing of treatment schedules respectively [37]. Similarly, in Malaysia, cancer patients who visited traditional medicine practitioners before seeking care at biomedical facilities experienced increased time to diagnosis and hence advanced stage cancers at diagnoses [38]. Our respondents thought that teaching T&CM in medical schools, and integration of the traditional and biomedical health systems would avoid delay in health seeking because patients would not have to attend either traditional medicine or biomedicine alternately but access both systems concurrently as the healthcare professionals would be aware and knowledgeable on T&CM and less likely to rebuke patients for attending care with traditional medicine practitioners. The complementary and synergistic situation described by our respondents is already being practiced in Canada and USA, where most patients use complementary therapies in addition to, not instead of, conventional care. Medical students in the integrative medicine centres in the USA and Canada are taught about complementary and traditional therapies so that they could be aware of commonly used traditional and complementary therapies, know how to talk to patients about such use, and know where to find/evaluate evidence about complementary therapies. In that regard, the medical students become physicians who could achieve the following important goals in their practices: Provide patient-centred care through careful considerations of the patient’s values, preferences and health goals, promote patient safety through thorough assessment of all the therapies a patient is using, and provide culturally competent care by considering patients’ values, culture and beliefs about disease aetiology and treatments that work for their illnesses.

Our findings that medical students are willing to learn more about T&CM is not isolated. A recent review revealed that medical students, especially those in pre-clinical years are enthusiastic to have T&CM theories and principles included in medical school curricula. The main reasons medical students want T&CM in their curricula include the need for awareness about T&CM, enable them communicate competently with their patients regarding T&CM use, and to enable them refer patients when appropriate to traditional health practitioners [39]. It is thus important to evaluate the feasibility for and consider including traditional and complementary medicine theories and principles into medical school curricula in Uganda and other low- and middle-income countries. Since the medical students themselves and their teachers are willing to embrace the concept of traditional and complementary medicine, it is likely that acceptability and adoption of the curricula change may not be a problem.

Lack of understanding of traditional medicine practices by healthcare professionals could undermine the ability of the doctors to advice patients regarding use or discontinuation of T&CM in particular circumstances. Patients have the right to self-determination and informed consent irrespective of whether they concern conventional medicine or T&CM. The healthcare professionals have the duty and obligation to inform them about the choices available for their illnesses based on best available evidence. Consequently, the patients need to be guided in their decisions so that they do not harm themselves. This brings into play the ethical principles of beneficence or commitment to social goal of upholding the public welfare and principle of non-maleficence [40]. For every patient, the healthcare professionals need to consider issues such as the severity and acuteness of the illness, the availability and quality of evidence of utility and safety of the desired T&CM treatment, the level of understanding of risks and benefits of the T&CM treatment combined with the patient’s knowing and voluntary acceptance of those risks, and the patient’s persistence of intention to use T&CM therapies. Irrespective of evidence of effectiveness and side effects of T&CM, healthcare professionals ought to fulfil their ethical obligations to protect the public from harm and maximize benefits from T&CM commonly used by their patients [41]. It is further argued that failure of healthcare professionals to advice patients against potentially harmful T&CM is unethical because such patients could get harmed by using the T&CM [42]. But the healthcare professionals cannot distinguish between the useful and potentially harmful T&CM if they do not engage with traditional and complementary medicine both at medical schools and through research. Policymakers therefore need to heed the WHO recommendations and follow the strategies for incorporating complementary and traditional medicines into medical school curricula and mainstream national health systems to promote safety of use of T&CM and increase accessibility to affordable and effective healthcare alternatives.

We found that teaching principles and practices of traditional and complementary medicine in the medical schools could offer an important opportunity for research on traditional medicines and determine evidence for their benefits and side effects. Research in locally available T&CM are a gateway to quality improvement and discovery of new medicines that could promote the health of the population. Support for research and development in T&CM has been low in most countries. Industries and governments have often put much less money into research into T&CM because of the prejudice by the policymakers that T&CM do not work and therefore not deserving of the taxpayers’ money [43]. Such prejudices are no more acceptable as it challenges the principle of social justice that would demand unfettered access to T&CM that have been demonstrated to be effective and safe. Healthcare professionals need to engage more vigorously in research involving T&CM in order to accumulate evidence for effectiveness and safety of T&CM for guiding informed consents and health care choices [40]. Healthcare professionals need to balance the ethical obligations to maximize good and avoid harm to their patients with the evidence of effectiveness and safety of both T&CM and conventional therapies. Awareness of T&CM and careful ethical considerations are necessary to provide holistic care for patients [14].

Several operational and ethical challenges were discussed regarding the introduction and teaching of T&CM principles and practices in medical schools in Uganda. These included few faculty to teach the subject, an already congested curricula, uncertainty regarding the intrinsic benefits and side effects of T&CM, obstruction by pharmaceutical companies, religious and personal values conflicts, and limited credible sources of information to support training on traditional medicines. These anticipated obstacles were viewed as bottlenecks that are surmountable and not reasons to disregard introducing T&CM theories, principles and practice into the medical school curricula in Uganda. This reiterates a common observation in human life that nothing important and serious comes by our way without challenges. To the contrary, anticipating and acknowledging challenges could serve a noble purpose of informing the development and strengthening of an evolving system. The issue of lack of evidence regarding the efficacy, effectiveness and side effects of T&CM emerged both as a reason for not teaching T&CM in medical schools and as a challenge that will likely increase the costs of teaching T&CM in medical schools because more researches will have to be carried out on local T&CM to inform evidence-base teaching. Research into and training in T&CM are indeed crucially needed as there is abundant evidence for inadequate knowledge regarding traditional medicines among biomedical healthcare professionals. For example, in a USA survey of 705 physicians in Colorado, only few physicians thought they had adequate knowledge about T&CM to allow them provide factual information to their patients for use or non-use. The majority of the surveyed physicians reported that they needed to learn more about T&CM in order to ably address patients’ concerns [44]. The self-confessed lack of awareness on T&CM among healthcare professionals could explain in part some of the perceptions that traditional medicines are not evidence-based practices and could inherently be harmful to the population. This was the case in Jordan, where up to 80 and 70% of Jordanian physicians reported that T&CM are not evidence-based and could cause harm to patients respectively [45]. Similarly, about 86% of 238 physicians in Finland were uncomfortable with use of alternative medicines for treatment of cancers majorly because of the perceived limited evidence on their benefits and specific side effects profiles. About half (51%) of these physicians believed that alternative therapies should not be used at all [46]. In light of how many cancer patients seek complementary therapies, and the need for evidence to guide care, the Society for Integrative Oncology was developed. Founded in 2003, the goal of the Society is to advance evidence-based comprehensive integrative health care to improve the lives of people affected with cancers [47].

This study has some limitations that potentially restrict the interpretations and transfer of our findings. Only one medical school, and traditional health practitioners in Kampala have been included in the study. The uniqueness of Makerere University School of Medicine including its position as the oldest and most famous medical school in the region could limit the utility of the findings. However, the national nature and universal mix of students and faculty at this university mean that opinion and experiences from all over the country and perhaps the East African region are represented. The T&CM practitioners in Kampala could be more enlightened and educated than practitioners outside the city. Therefore, the views here could be somewhat different from those in the upcountry places. A larger sample including T&CM practitioners from all over the country and other medical schools could strengthen the evidence for the evaluation of the need for including T&CM into the medical school curricula and integration of T&CM into the national health system. Second, the use of qualitative methods to investigate this matter is excellent in exposing the depth of ideas and opinions of key stakeholders but falls short of assessing the breath and proportions of stakeholders that would support or resist the idea of including T&CM in the medical school curricula. While deeper understanding of the nuances involved in introducing a new concept into the curricula is important, acceptability of the concept equally depends on the proportion of stakeholders who share in the concept.

## Conclusion

Integration of biomedical and traditional medicine practices is considered reasonable, feasible and beneficial to population health outcomes. Training medical students on concepts and principles of T&CM could enhance their understanding of the traditional health systems and foster integration. However, there are operational and ethical challenges that need to be further evaluated and feasible solutions generated before actual introduction of traditional and complementary medicine theories and principles into the medical school curricula.

## Additional files

Additional file 1: Focus group discussions guide for medical students. (DOCX 37 kb)

Additional file 2: In-depth interview guide for Medical School Lecturers, and traditional and complementary medicine practitioners. (DOCX 40 kb)
