# Supplementary material for: Turning a blind eye and a deaf ear to traditional and complementary medicine practice does not make it go away: a qualitative study exploring perceptions and attitudes of stakeholders towards the integration of traditional and complementary medicine into medical school curriculum in Uganda
Source: BMC Med Educ. 2018 Dec 18;18:310. doi: 10.1186/s12909-018-1419-4 (PMC6299601; doi:10.1186/s12909-018-1419-4)
Supplement: Supplementary file 1 — Focus group discussions guide for medical students. (DOCX 37 kb) [file 12909_2018_1419_MOESM1_ESM.docx]

# Focus group discussions guide for medical students

**INTEGRATING TRADITIONAL AND COMPLEMENTARY MEDICINE IN MEDICAL SCHOOL CURRICULA IN UGANDA**

**Specific objective:**

To explore the perceptions and attitudes of second and fifth year medical students at the College of Health Sciences Makerere University regarding inclusion of traditional and complementary medicine (T&CM) theories and fundamentals into medical school curricula in Uganda.

**Participants:** Second and fifth year medical students

**Time:** The interview is expected to last 45 to 90 minutes.

**Informed consent:** Purpose of interview will be explained to participants and signed informed consent sought before interview.

**Venue:** A quiet room or open space to ensure openness and avoid interferences.

**Recordings:** Audio recordings will be done to augment field notes.

**Introduction:** In this study we aim to assess the feasibility and acceptability of integration of Traditional and Complementary Medicine principles and practices into medical school curricula as a step towards the integration of T&CM with mainstream healthcare system in Uganda. Knowledge from this study can provide guidance on the training needs of physicians and traditional and complementary medicine practitioners which may enhance the integration process.

**Section A: Sociodemographic characteristics and training achievements**

a) Study number: ………………………………………………….

b) Year of study: …………………………………………………..

c) Age range: ………………………………………………….

d) Gender: ……………………………………………….

**Section B: Study Guide**

**Theme 1. Definition of traditional and complementary medicines (T&CM).**

*Probes*:

a) Kindly share with us what you consider an appropriate definition of traditional and complementary medicine.

**Theme 2: Integration of traditional and complementary medicine into medical school curricula.**

***Probes:***

a) What is your opinion on introducing the theories, principles and practices of T&CM into the undergraduate curricula in Uganda? Why or why not include?

b) Are there some ethical concerns you have with including T&CM in the undergraduate curricula?

(c) What ethical principles are you concern with and why?

(d) If you consider adding T&CM into the undergraduate curricula a well thought process – what are the goals or aims you consider most important for adding T&CM into the curricula? Please discuss as many goals as you consider important: In otherward, why should T&CM be taught in medical school?

(e) If T&CM is to be taught as an undergraduate course, at what level or year of study should it be taught and why?

(f) What methods of teaching or training could be adopted for T&CM?

(g) Who should teach T&CM and why?

(h) What are some of the challenges or constraints you could anticipate regarding including T&CM course into the undergraduate curricula in medical schools in Uganda? Please be as exhaustive as possible in our approach to the challenges (e.g. Time constraints/curricula congested already, lack of teachers).

(i) What are some of the gains or benefits of including T&CM in the undergraduate curricula in medical schools in Uganda?

(j) What could be some of the dangers or risks of not teaching undergraduate medical students on theories, principles and practices of T&CM? Please discuss exhaustively.

**Theme 3: Sources of information about T&CM (For healthcare professionals only)**

***Probes*:**

a) What are your most common sources of information about traditional, traditional and complementary medicines?

b) Please discuss any particular difficulties you experience when seeking information about traditional, traditional and complementary medicines.

**Thank you for participating**
