# Supplementary material for: Turning a blind eye and a deaf ear to traditional and complementary medicine practice does not make it go away: a qualitative study exploring perceptions and attitudes of stakeholders towards the integration of traditional and complementary medicine into medical school curriculum in Uganda
Source: BMC Med Educ. 2018 Dec 18;18:310. doi: 10.1186/s12909-018-1419-4 (PMC6299601; doi:10.1186/s12909-018-1419-4)
Supplement: Supplementary file 2 — In-depth interview guide for Medical School Lecturers, and traditional and complementary medicine practitioners. (DOCX 40 kb) [file 12909_2018_1419_MOESM2_ESM.docx]

# In-depth interview guide for Medical School Lecturers and traditional and complementary medicine practitioners

**INTEGRATING TRADITIONAL AND COMPLEMENTARY MEDICINE IN MEDICAL SCHOOL CURRICULA IN UGANDA**

**Specific objectives:**

(a) To explore the perceptions and attitudes of the lecturers and professors in the departments of paediatrics and internal medicine in the College of Health Sciences Makerere University regarding inclusion of traditional and complementary medicine (T&CM) theories and fundamentals into medical school curricula in Uganda.

(b) To explore the perceptions and attitudes of leaders of traditional health practitioners associations in Kampala regarding inclusion of traditional and complementary medicine (T&CM) theories and fundamentals into medical school curricula in Uganda.

**Participants:**

(i) Lecturers and Professors at the departments of medicine, psychiatry and paediatrics.

(ii) Leaders of traditional and complementary medicine practitioners association resident in Kampala at the national associations of traditional healers.

**Time:** The interview is expected to last 45 to 90 minutes.

**Informed consent:** Purpose of interview will be explained to participants and signed informed consent sought before interview.

**Venue:** A quiet room or open space to ensure openness and avoid interferences.

**Recordings:** Audio recordings will be done to augment field notes.

**Introduction:** In this study we aim to assess the feasibility and acceptability of integration of Traditional and Complementary Medicine principles and practices into medical school curricula as a step towards the integration of T&CM with mainstream healthcare system in Uganda. Knowledge from this study can provide guidance on the training needs of physicians and traditional and complementary medicine practitioners which may enhance the integration process.

**Section A: Sociodemographic characteristics and training achievements**

a) Study number: ………………………………………………….

b) Qualifications: ……………………………………………………………………………………………………………………………………………………………………………………………………………………………………………………………………………………………………………………

(Please list all health and non-health related formal trainings leading to awards of certificates, diplomas, degrees and fellowships).

c) Status in employment (in service/retired): ……………………………………………..

d) Employer: …………………………………………………………………..

e) Age: ………………………………………………….

f) Gender: ……………………………………………….

g) Religion: ……………………………………………..

h) Marital status: ………………………………………..

i) Ever prescribed traditional and complementary medicine in recent 3 months

Yes: ………..

No: …………

**Section B: Study Guide**

**Theme 1. Definition of traditional and complementary medicines (T&CM).**

*Probes*:

a) Kindly share with us what you consider an appropriate definition of traditional and complementary medicine.

b) Who then is a T&CM practitioner?

**Theme 2: Integration of traditional and complementary medicine into medical school curricula.**

***Probes:***

a) What is your opinion on introducing the theories, principles and practices of T&CM into the undergraduate curricula in Uganda? Why or why not include?

b) Are there some ethical concerns you have with including T&CM in the undergraduate curricula?

(c) What ethical principles are you concern with and why?

(d) If you consider adding T&CM into the undergraduate curricula a well thought process – what are the goals or aims you consider most important for adding T&CM into the curricula? Please discuss as many goals as you consider important: In otherward, why should T&CM be taught in medical school?

(e) If T&CM is to be taught as an undergraduate course, at what level or year of study should it be taught and why?

(f) What methods of teaching or training could be adopted for ICM?

(g) Who should teach T&CM and why?

(h) What are some of the challenges or constraints you could anticipate regarding including T&CM course into the undergraduate curricula in medical schools in Uganda? Please be as exhaustive as possible in our approach to the challenges (e.g. Time constraints/curricula congested already, lack of teachers).

(i) What are some of the gains or benefits of including T&CM in the undergraduate curricula in medical schools in Uganda?

(j) What could be some of the dangers or risks of not teaching undergraduate medical students on theories, principles and practices of T&CM? Please discuss exhaustively.

**Theme 3: Integration of traditional and complementary medicine with biomedicine.**

***Probes:***

a) What is your opinion on introducing the use of T&CM/herbal medicines at the formal health facilities? Why or why not?

b) What could be some of the benefits of integrating T&CM with biomedicine? (List all perceived benefits – e.g. integration could attract more patients to visit the health facilities once sick and that would provide an opportunity for early detection and treatment by healthcare professionals).

c) What are some of the dangers or potential dangers of integrating T&CM with biomedicine?

d) How should collaborations between T&CM and biomedicine be achieved? How should the two come to work together?

(**Further probes:** Build a traditional, traditional and complementary medicine and practitioners training institute to: train traditional healers on aspects of biomedicine so that traditional healers may become partners in health education, participate in disease prevention, early recognition and referral of patients to health facilities, and provide in-service training for healthcare professionals at the Institute for T&CM).

**Theme 4: Sources of information about T&CM (For healthcare professionals only)**

***Probes*:**

a) What are your most common sources of information about traditional and complementary medicines?

b) Please discuss any particular difficulties you experience when seeking information about traditional and complementary medicines.

**Thank you for your cooperation and time**
